# Osteomyelitis of the Third Metatarsal Mimicking a Neoplastic Lesion Due to a Retained Wooden Foreign Body

**DOI:** 10.7759/cureus.100642

**Published:** 2026-01-02

**Authors:** Yahya Mohamed Faqihi, Ibrahim M. Mahmoud, Sayyaf Abdullah Najmi, Elsadig Ibrahim Arbab, Mohamed Elsaid Aboushady, Ibrahim Fadl Mahmoud

**Affiliations:** 1 Department of Orthopedic Surgery, Armed Forces Hospital Jazan, Jazan, SAU; 2 Department of Radiodiagnosis and Medical Imaging, Armed Forces Hospital Jazan, Jazan, SAU; 3 Department of Anesthesia, Intensive Care and Pain Management, Damietta Faculty of Medicine, Al-Azhar University, New Damietta, EGY; 4 Department of Anesthesiology and Intensive Care, Armed Forces Hospital Jazan, Jazan, SAU

**Keywords:** computed tomography, diagnostic delay, foot, foreign body, magnetic resonance imaging, osteomyelitis, pediatric, puncture wound, wood

## Abstract

Plantar puncture wounds are a common entity among children, particularly in areas where walking barefoot is common, but the detection of radiolucent foreign bodies (FBs), like those made of wood, remains elusive, with the risk of delayed diagnosis. The patient was a four-year-old girl who presented with chronic foot pain, limping, and a localized dorsal mass six months following a plantar puncture injury. Initial management at another facility consisted of exploration for a suspected FB and abscess, guided by an ultrasound report and an attempt at removal guided by ultrasound; however, her symptoms persisted. Plain radiographs showed osteolysis and sclerosis of the third metatarsal, which would be suspicious for osteomyelitis or a neoplastic process. Computed tomography (CT) and magnetic resonance imaging (MRI) subsequently provided a clear identification of a 2-cm retained wooden FB eroding into the medullary canal of the third metatarsal. The FB was successfully removed via surgical exploration through a dorsal approach, followed by debridement and curettage. This case underlines the limitations of plain radiographs and ultrasound in detecting radiolucent FBs. Early application of advanced cross-sectional imaging studies, such as CT and MRI, is indispensable in penetrating trauma with organic material in order to avoid delayed diagnosis and grave complications such as osteomyelitis.

## Introduction

Plantar puncture wounds are the most common mechanism for foreign body (FB)-related foot trauma in children [1]. Frequently encountered FBs include needles, glass, and organic materials such as thorns and wooden splinters. While metallic FBs are radiopaque and easily visualized on plain radiographs, organic materials are radiolucent, which makes their diagnosis particularly challenging [2].

Initial management typically consists of thorough wound irrigation and exploration. However, deep-seated or radiolucent FBs are frequently missed, resulting in delayed presentation with complications such as cellulitis, abscess formation, chronic sinuses, and osteomyelitis [3]. The subsequent inflammatory and infectious processes may induce bony changes that mimic neoplastic lesions, further complicating diagnosis. This issue is particularly problematic in pediatric patients, where a history of trauma may be unclear or forgotten [4-10]. Escalation from X-ray or ultrasound to CT or MRI is done when symptoms persist despite initial management. CT is effective for identifying bony erosion and often detects wood, which appears as a linear hyperdensity. On MRI, the FB typically presents as a linear signal void [2,3].

The objective of this case report is to highlight the diagnostic challenges associated with retained radiolucent FBs and to emphasize the essential role of advanced imaging and meticulous surgical planning in preventing long-term morbidity.

## Case presentation

A four-year-old female patient with obesity (30 kg) was referred to our orthopedic clinic with a six-month history of intermittent pain and a newly developed, localized mass on the dorsal aspect of her right foot, limping, no redness or discharge. History revealed a plantar puncture wound from a wooden object six months prior. Five months before presentation, she had undergone surgical exploration in another hospital via a plantar approach between the third and fourth metatarsal heads for a suspected FB and abscess, guided by an ultrasound report. A postoperative ultrasound performed one month later showed no residual FB. Despite this, her symptoms persisted.

Upon presentation, physical examination revealed a tender, localized mass on the dorsal aspect of the third metatarsal. There was no erythema, color, sinus tract, or drainage. Neurovascular status was intact. Laboratory investigations were within normal limits, including a white blood cell count of 4,500 cells/mm³ (reference range: 4,000-11,000 cells/mm³), a C-reactive protein level of <6 mg/L (reference: < 6 mg/L), and an erythrocyte sedimentation rate of 6 mm/hr (reference: 8 mm/hr).

**Figure 1:**
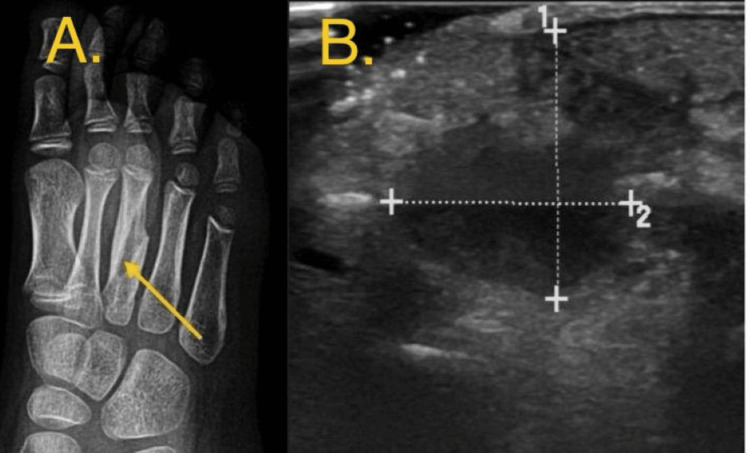
A: Pre-operative oblique radiograph of the foot demonstrating chronic osteolysis, tunneling & relative sclerosis of the third metatarsal shaft. B: Pre-operative ultrasound image showing an irregular hypoechoic collection showing internal echoes within, yet no sizable sonopaque foreign bodies could be appreciated

Plain radiography of the foot demonstrated osteolysis and osteosclerosis of the third metatarsal shaft, highly suspicious for chronic osteomyelitis or a benign bone neoplasm (Figure 1). However, these findings can be misleading. Differential diagnosis was Ewing sarcoma, Langerhans cell histiocytosis, chronic osteomyelitis, and Brodie’s abscess, emphasizing the need for cautious interpretation and awareness of the limitations of this imaging modality. 

**Figure 2:**
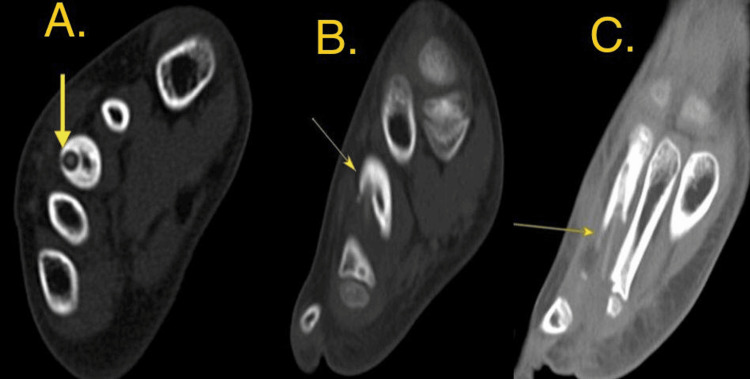
CT image: Slice thickness and reconstruction (0.8 mm) The long faintly dense structure (foreign body) density is around (150 HU) seen partially emerging into and surrounded by dense cortex with consequent narrowing of the medullary canal while its distal portion is seen extra osseous. (A: axial view, B: sagittal view & C: coronal view) Chronic medullary sclerosis & tunneling harboring a relatively dense, elongated foreign body (arrow) within the medullary canal of the third metatarsal, with associated bone destruction.

**Figure 3:**
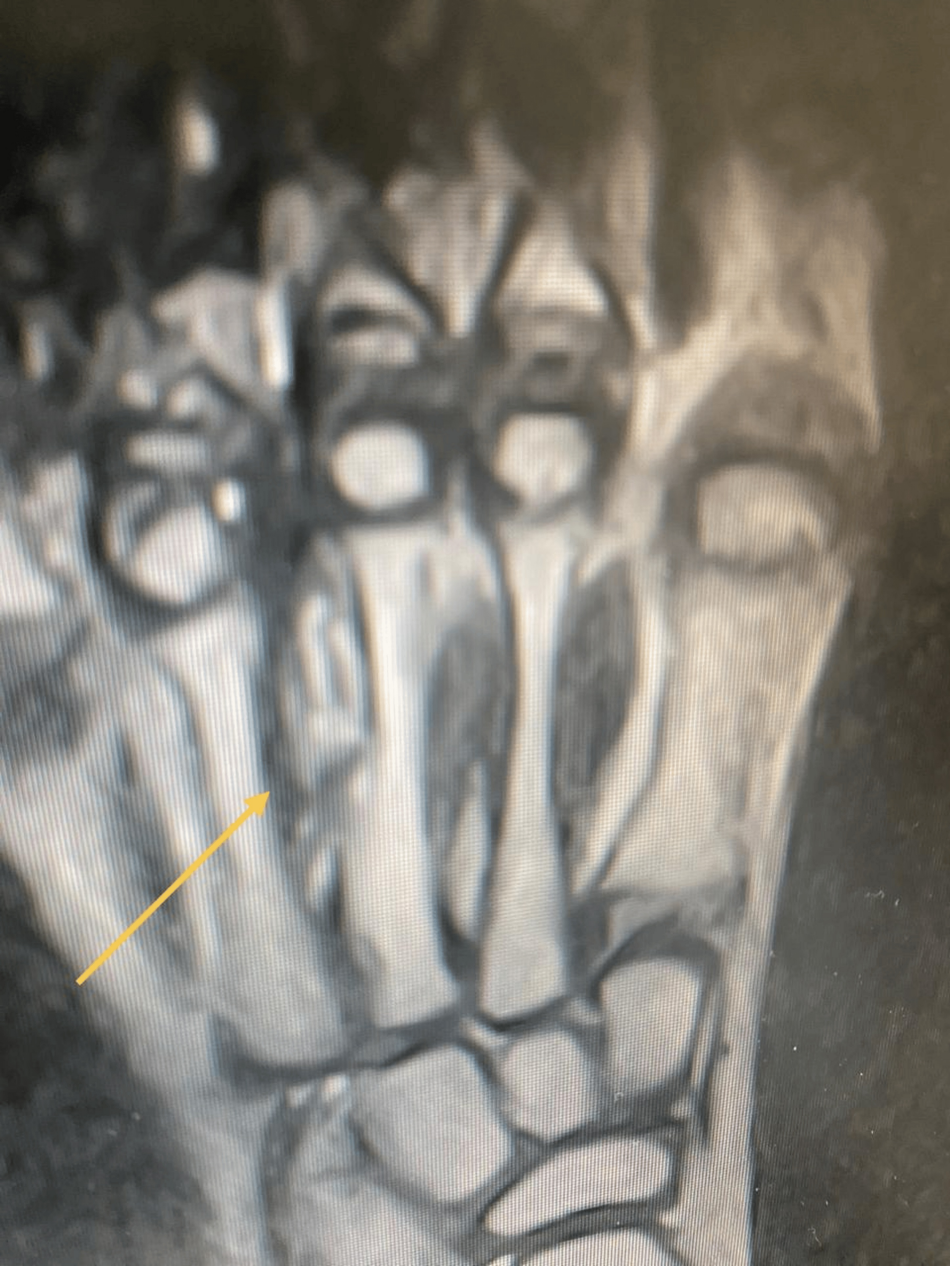
Rt foot MRI. Multi-planner MRI for the right foot with coronal PD FS, sagittal T1, PD FS, axial T2 and PD FS was performed. Limited study with poor quality at the third metatarsal bone; the mid portion shows evident cortical thickening and mild soft tissue edema. PD FS: Proton density fat saturation

To better characterize the lesion, advanced imaging with CT and MRI was performed, which enabled detailed visualization of the FB and associated changes. The CT scan clearly identified a linear, hyperdense FB within the medullary canal of the third metatarsal, associated with cortical destruction and an adjacent soft-tissue abscess (Figure 2). The MRI confirmed a 2 cm linear signal void on all sequences, consistent with a wooden FB, surrounded by extensive bone marrow edema and soft-tissue inflammation (Figure 3).

**Figure 4:**
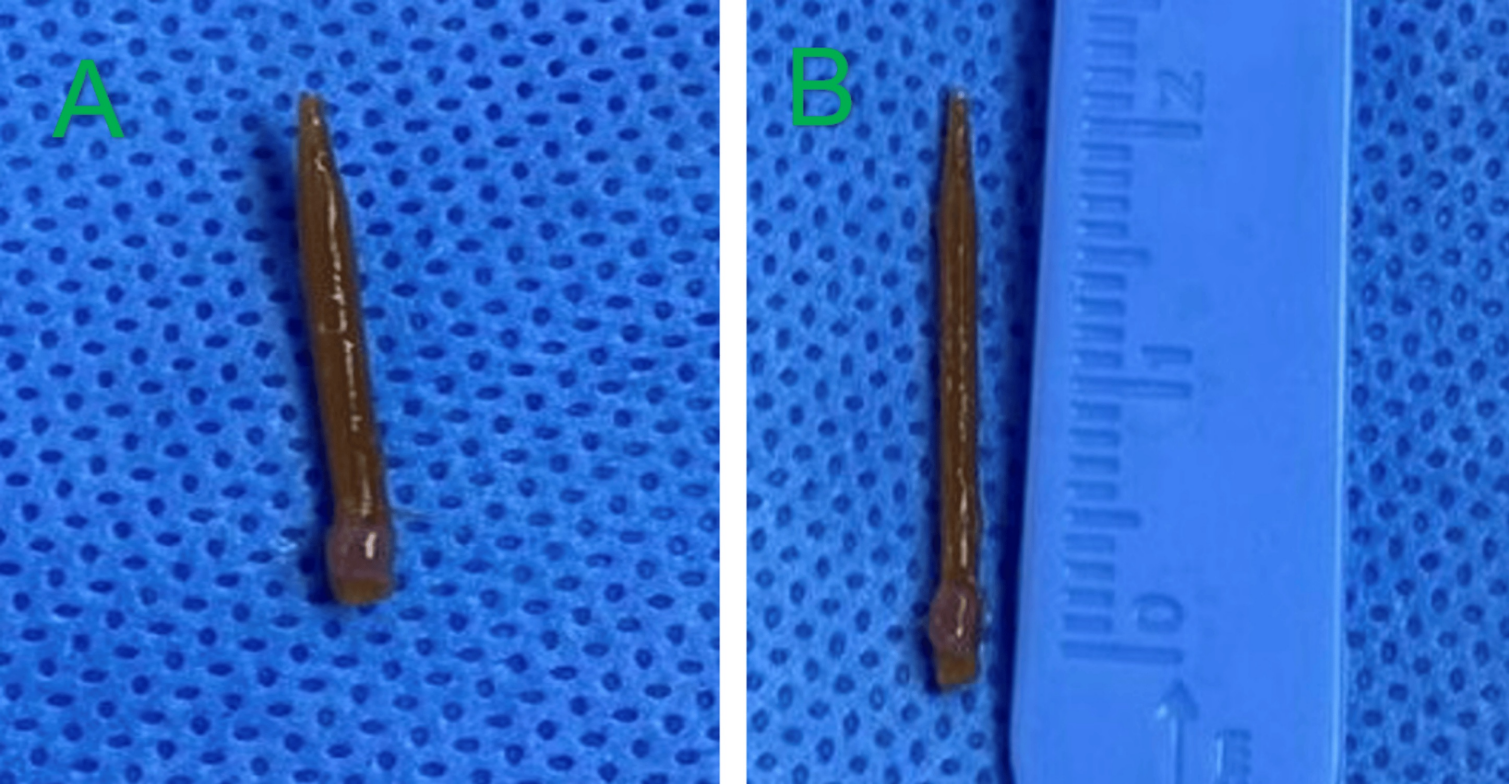
Photograph of the retrieved wooden foreign body, measuring approximately 2 cm in length.

The patient was taken to the operating room for surgical exploration. A dorsal approach to the third metatarsal was utilized. Intraoperatively, a wooden piece was found adjacent to the bone, tracking into the medullary canal through a cortical defect near the base of the metatarsal. The FB was successfully removed (Figure 4), and thorough debridement and curettage of the involved bone and soft tissues were performed. Intraoperative cultures were obtained; however, no bacterial growth was observed after five days of incubation. Histopathology revealed chronic inflammatory changes consistent with osteomyelitis with no evidence of malignancy (necrotic bone spicules, chronic inflammatory granulation tissue with plasma cells and lymphocytes, and marrow fibrosis). Additional anaerobic cultures were submitted and incubated for an extended period (14 days). Preoperative antibiotic administration may have contributed to the negative culture results.

Postoperatively, the patient was immobilized in a back slab and received intravenous Cefazolin 1 g three times daily (100 mg/kg/day) for five days, followed by oral cephalexin 250 mg four times daily (30 mg/kg/day) for three weeks. The wound healed without complication.

**Figure 5:**
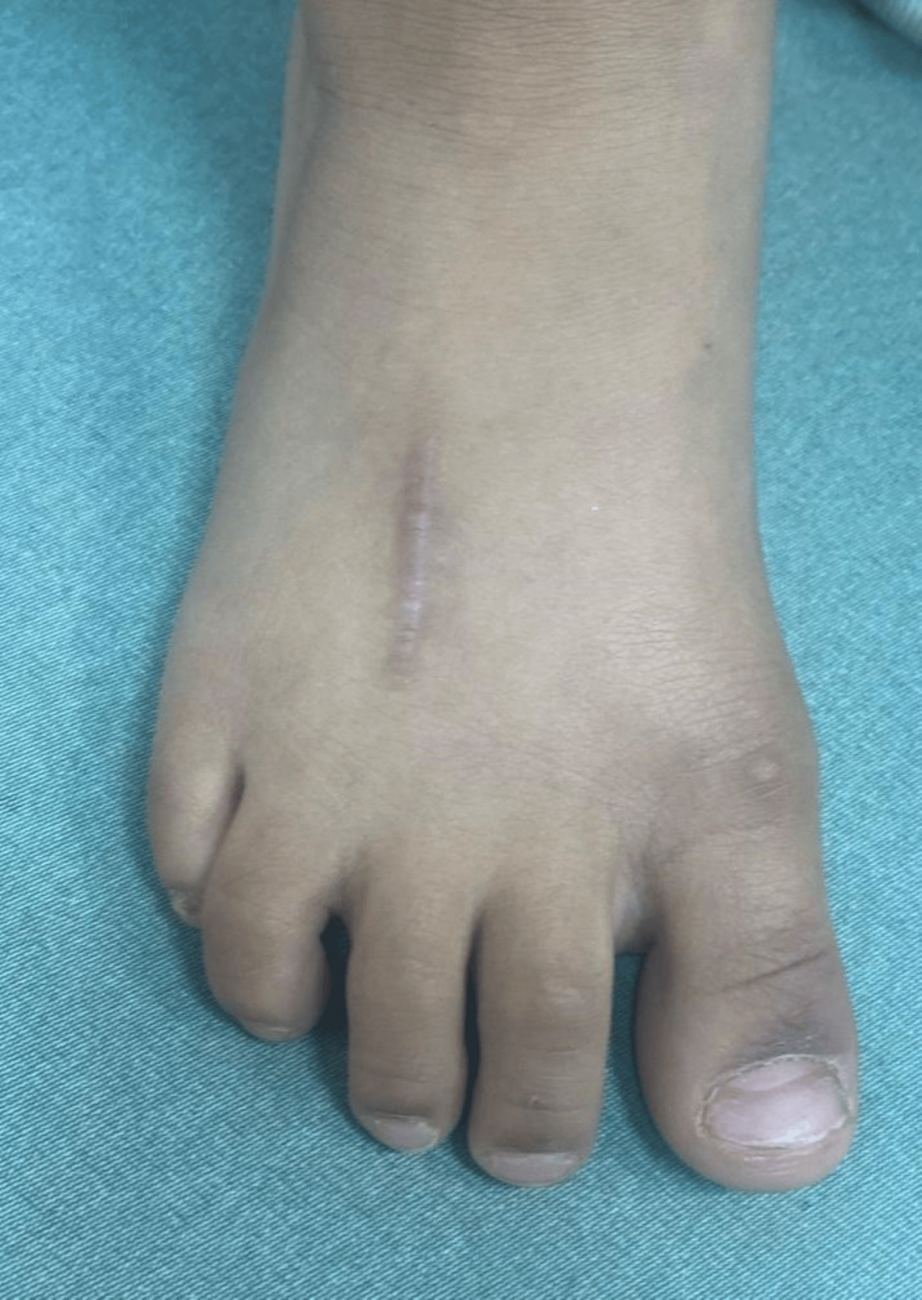
Site of incision with a healed scar and no signs of infection.

At the six-month follow-up, the patient was asymptomatic and ambulating normally without a limp (Figure 5).

**Figure 6:**
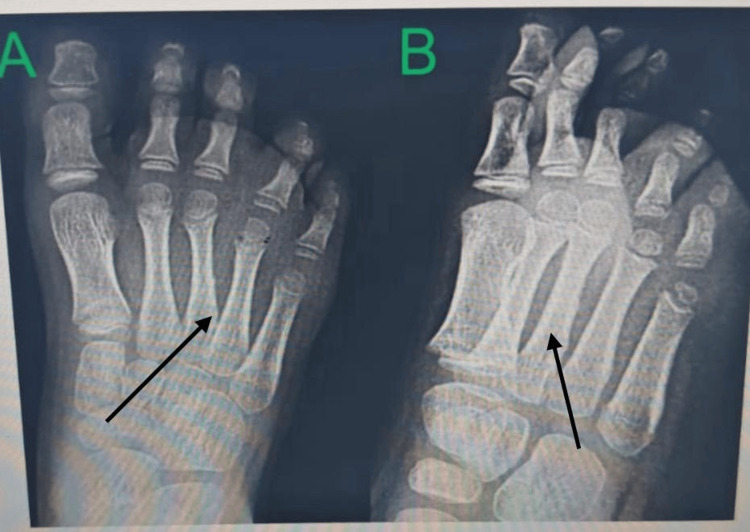
Post-operative radiograph at the six-month follow-up of foot (A: anteroposterior and B: oblique view), showing complete healing of the third metatarsal with resolution of osteolytic changes.

Follow-up radiographs demonstrated complete resolution of the osteolytic changes and healing of the third metatarsal, with restoration of normal cortical and medullary architecture (Figure 6).

## Discussion

Retained FBs (such as wood, rubber, or animal spines) can induce chronic infection and inflammatory changes in bone, leading to osteolytic lesions that are radiographically and clinically indistinguishable from osteomyelitis or even bone tumors [5-10].

Symptoms of delayed diagnosis may be nonspecific (pain, swelling, draining sinus), and standard X-rays may miss radiolucent materials such as wood or rubber. MRI and CT are more sensitive for detecting both the FB and associated bone changes [5-8].

This case exemplifies a frequently overlooked scenario in pediatric orthopedic trauma, in which a retained radiolucent FB results in significant bone infection. It may also occur in adults; however, it is more frequently reported in the pediatric population, likely due to factors such as unclear clinical history, subtle initial symptoms, and variations in bone physiology. The initial diagnostic and therapeutic errors offer important lessons for clinical practice.

The primary challenge lies in the inherent properties of organic FBs. Wood was not visible on plain radiographs, which revealed only the secondary bony reaction months later. Ultrasound, while helpful in detecting superficial soft-tissue FBs and collections, is highly operator-dependent and has limited ability to visualize FBs embedded in or eroding into bone, as demonstrated by the false-negative postoperative scan in our case [2,4].

CT and MRI play an important and definitive role in such complex cases. CT is excellent for delineating bony erosion and can often detect wood, which usually appears as a linear hyperdensity [2]. MRI is superior for delineation of the extensive soft-tissue and bone marrow edema pattern, while the FB itself shows characteristic linear signal voiding, thus providing a clear roadmap for surgical intervention [3].

The negative intraoperative cultures are a notable feature, which is not uncommon in FB-related osteomyelitis, particularly when the FB is organic. The pathologic process can be primarily an inflammatory reaction to the retained organic material, which secondarily creates a devitalized environment susceptible to infection. The diagnosis in such cases was often based on histopathological findings of acute and chronic inflammation rather than on positive microbiological results [11,12].

From a surgical perspective, this case illustrates the importance of approach selection from the surgical perspective. Initial failure through the plantar approach, likely the entry site, serves to emphasize that a surgical corridor should be planned based on the final position of the FB as elucidated by cross-sectional imaging, and not just the point of penetration. The definitive dorsal approach afforded a direct route to both the FB and the involved metatarsal, which made complete removal and debridement possible [13,14].

## Conclusions

Retained wooden FBs in the foot can cause complicated clinical manifestations that frequently mimic severe bone diseases like neoplastic lesions or osteomyelitis. Both ultrasonography and plain radiography have significant drawbacks. Since wood is usually radiolucent, it cannot be seen on an X-ray. Although ultrasonography is capable of detecting surface FBs, its accuracy is largely operator-dependent and decreases when gas, inflammation, or deeper objects are present. Physicians need to keep a high level of suspicion, especially when symptoms persist after starting treatment. Early use of MRI or CT allows for precise diagnosis and efficient surgical planning, ensures total removal of FBs, avoids long-term complications, and restores limb function. For foot injuries with unusual or persistent symptoms, early CT or MRI should be considered to prevent severe complications and improve patient outcomes.
